# Carbon-assisted growth and high visible-light optical reflectivity of amorphous silicon oxynitride nanowires

**DOI:** 10.1186/1556-276X-6-469

**Published:** 2011-07-25

**Authors:** Lei Zhang, Tielin Shi, Zirong Tang, Dan Liu, Shuang Xi, Xiaoping Li, Wuxing Lai

**Affiliations:** 1 State Key laboratory of Digital Manufacturing Equipment and Technology, Huazhong University of Science and Technology, Wuhan 430074, China; 2 Wuhan National Laboratory for Optoelectronics, Huazhong University of Science and Technology, Wuhan 430074, China

## Abstract

Large amounts of amorphous silicon oxynitride nanowires have been synthesized on silicon wafer through carbon-assisted vapor-solid growth avoiding the contamination from metallic catalysts. These nanowires have the length of up to 100 μm, with a diameter ranging from 50 to 150 nm. Around 3-nm-sized nanostructures are observed to be homogeneously distributed within a nanowire cross-section matrix. The unique configuration might determine the growth of ternary amorphous structure and its special splitting behavior. Optical properties of the nanowires have also been investigated. The obtained nanowires were attractive for their exceptional whiteness, perceived brightness, and optical brilliance. These nanowires display greatly enhanced reflection over the whole visible wavelength, with more than 80% of light reflected on most of the wavelength ranging from 400 to 700 nm and the lowest reflectivity exceeding 70%, exhibiting performance superior to that of the reported white beetle. Intense visible photoluminescence is also observed over a broad spectrum ranging from 320 to 500 nm with two shoulders centered at around 444 and 468 nm, respectively.

## Introduction

Silicon oxynitride (Si-O-N) materials have received considerable attention due to their special physical, chemical, and electrical properties [1-4]. Compositionally and structurally, silicon oxynitride can be regarded as the transition from silicon oxide to silicon nitride. Many of its physical properties also display a high extent of flexibility between the two extremes, changing continuously with N/O ratio [5]. For example, the Si-O-N film possesses a large range of refractive indices spanning from 1.45 to 2.00. Moreover, the Si-O-N layers also show a high degree of optical transparency in the visible and near infrared spectral regions, which enables a variety of optical designs for integrated optics applications [6-10]. On the other hand, nanowires have intrigued considerable research enthusiasm for their unique physical properties and promising application as building blocks in nanoscale electronics and optoelectronics [11]. Therefore, a controlled synthesis of silicon oxynitride nanowires deserves intense research attention.

However, reports on Si-O-N nanowires were so far rather rare [12-16]. The reported synthesis processes often involved the utilization of transition metals as catalysts in quartz tube furnace for pyrolysis, and sometimes inductively coupled coil was applied to obtain NH_3 _plasma for the nanowire growth [12-15]. These methods are unfavorable due to either the metal contamination to the resulted nanowires or the complicated equipment. Up to now, the optical properties of the Si-O-N nanowires remain largely unexplored, with only blue photoluminescence property recorded in literature [13,16]. In this letter, we develop an inexpensive, easy, repeatable, and catalyst-free method to obtain a kind of amorphous Si-O-N nanowire showing high optical reflectivity in visible-light wavelength, and investigate its growth mechanism.

### Experimental approach

In a typical synthesis procedure, an amorphous carbon film was first sputtered on a single-crystal Si wafer (1 0 0) in spraying etching instrument (SCD050, Faraday Technology, Clayton, OH, USA). Secondly, the resulting silicon substrate was loaded to an alumina crucible boat, placed inside a quartz tube furnace. After the furnace was evacuated to 10^-3 ^Torr, H_2 _(5%)/N_2 _mixed gas flow was kept through the tube at the rate of 2,000 sccm. The crucible was heated up to 1,200°C with a ramping rate of 15°C/min. After being maintained in 1,200°C for 4 h, the furnace was naturally cooled down to room temperature, and white products (later found to be Si-O-N nanowires) were found on the Si wafer.

Finally, the morphologies of these white products were characterized by scanning electron microscopy (SEM, Quanta 200, FEI Company, Hillsboro, OR, USA). Please check, high-resolution transmission electron microscopy (HRTEM, Tecnai 12, FEI Company, Hillsboro, OR, USA) equipped with an energy-dispersive X-ray (EDX), and high-angle annular dark field (HAADF) scanning transmission electron microscopy (STEM, Tecnai G2 F30 S-TWIN, FEI Company, Hillsboro, OR, USA). Chemical composition analysis was investigated by X-ray photoelectron spectroscopy (XPS, Shimadzu/Kratos AXIS Ultra DLD, Kratos Analytical, Chestnut Ridge, NY, USA), equipped with a standard and monochromatic source (Al Kα) operated at 150 W. The optical reflectivity of these nanowires was also studied using a Datacolor Elrepho photospectrometer (Datacolor ELREPHO, Lawrenceville, NJ, USA), and their photoluminescence (PL) measurement was conducted at room temperature using an FP-6500 with Xenon lamp line (Jasco, Essex, UK) of 258 nm as the excitation source.

## Results and discussion

**Figure 1:**
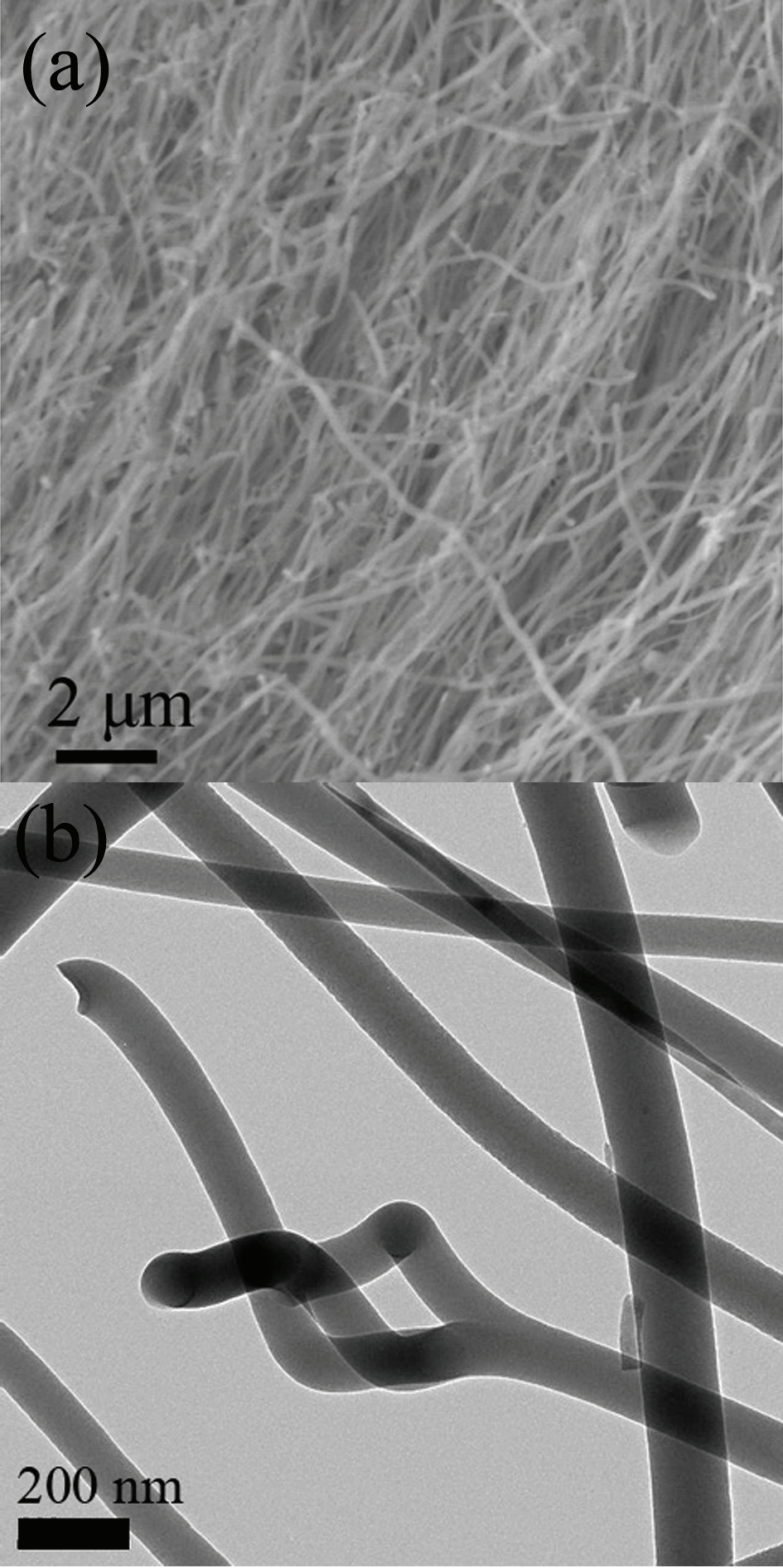
**SEM and TEM images of Si-O-N nanowires**. **(a) **A typical SEM image of the as-grown Si-O-N nanowires. **(b) **A typical TEM image of Si-O-N nanowires.

Figure 1a, b shows the typical SEM and TEM images of nanowires on a Si substrate, respectively. The diameter of the nanowires is ranging from 50 to 150 nm, and the length is about 100 μm. Splitting phenomena of nanowires are observed, where a three-branch structure with the same diameter is demonstrated in Figure 1b. The observation is different from the report by L. Gu *et al*., in which the diameter of the splitted branches is usually smaller than that of the trunk [15]. The individual branches here can be differentially chemically functionalized and terminated to create complex multiple chemical sensors in one unit [17].

**Figure 2:**
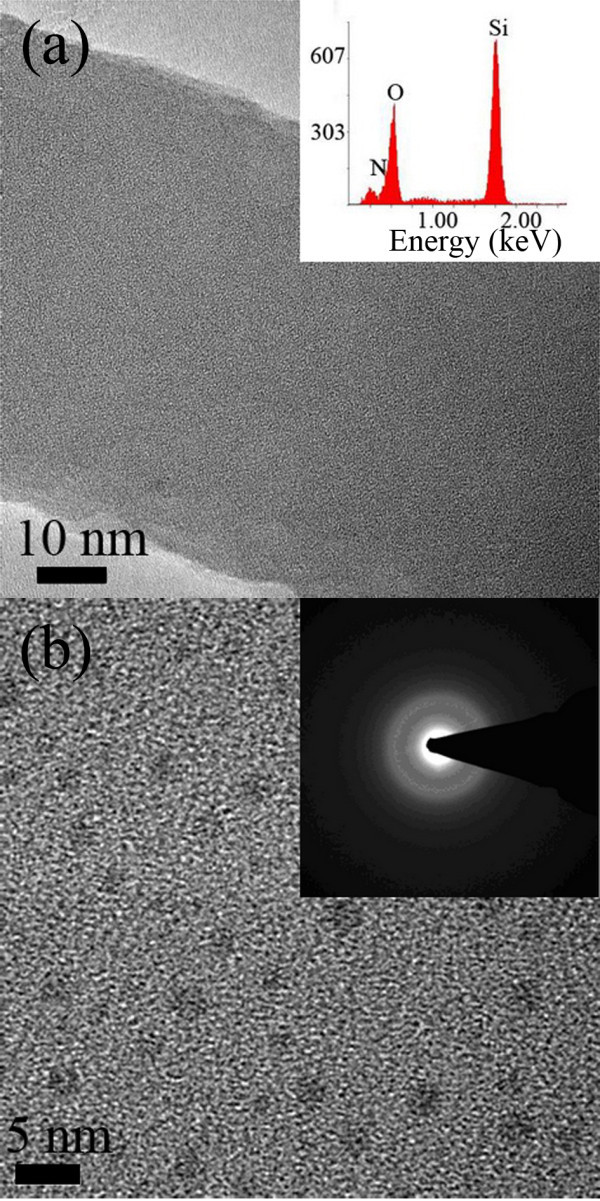
**TEM images of the surface and cross-section morphologies of a nanowire**. **(a) **TEM image of a Si-O-N nanowire and the corresponding EDX analyses as an inset. **(b) **An HRTEM image of the nanowire's cross-section and the corresponding SAED patterns as an insert.

Figure 2a, b shows typical TEM images of the surface and cross-section morphologies of a nanowire, respectively, indicating that the resulting nanowires are amorphous. The corresponding selected area electron diffraction (SAED) pattern confirms its amorphous structure shown as an inset in Figure 2b. EDX analyses shown as an inset in Figure 2a revealed that the chemical composition of the nanowire consists basically of three elements of Si, N, and O, with the ratio of approximately 2.75:1:3.60. We observed that there were homogeneously distributed nanostructures in the cross-section of the Si-O-N nanowire matrix, with a diameter of about 3 nm, shown as dark spots in Figure 2b. A similar observation was reported by D. Criado *et al*. in the Si-O-N film study [18]. Their study showed that the homogeneously distributed nanostructures can be found in SiO_2_-like Si-O-N samples, where the dark spot area might be the structure related with N element. In our study, the kind of distribution is observed in all cross-sections of Si-O-N nanowires, but none is obtained on the surface. These unique configurations might determine the growth behavior of multi-element amorphous nanowires and lead to the splitting phenomena where the branches and the trunk have the same diameter.

**Figure 3:**
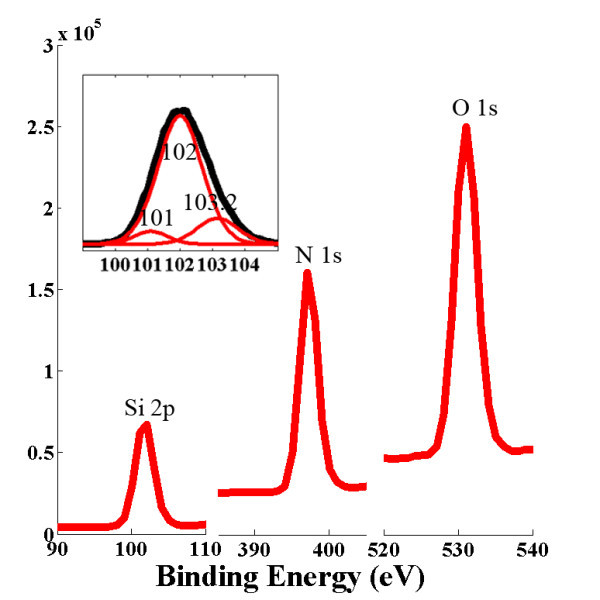
**XPS spectra of the as-synthesized nanowire bundles**. Binding energies of the Si 2*p *are shown as an insert.

The chemical composition of the nanowires was further characterized by XPS. Figure 3 shows the XPS spectra of the resulting nanowires with Si 2*p*, N 1*s*, and O 1*s *signals, with the binding energies of the Si 2*p *as an insert. Three strong XPS signals confirm that the nanowire is composed of the three elements (Si, O, and N). The Si 2*p *spectrum is decomposed into three Gaussian peaks located at 101, 102, and 103.2 eV. The two peaks at 101 and 103.2 eV are attributed to Si-N and Si-O bonds, respectively. The peak at 102 eV can be attributed to the Si-CH_x _bonds which may be due to organic gas adsorption. From the integrated areas of the Si 2*p*, N 1*s*, and O 1*s *peaks, it is estimated that the Si, N, and O atoms of the nanowires have the ratio of approximately 0.70:1:1.28. The apparent ratio difference of the three elements between EDX and XPS indicates that N element concentration is much higher at the surface area.

**Figure 4:**
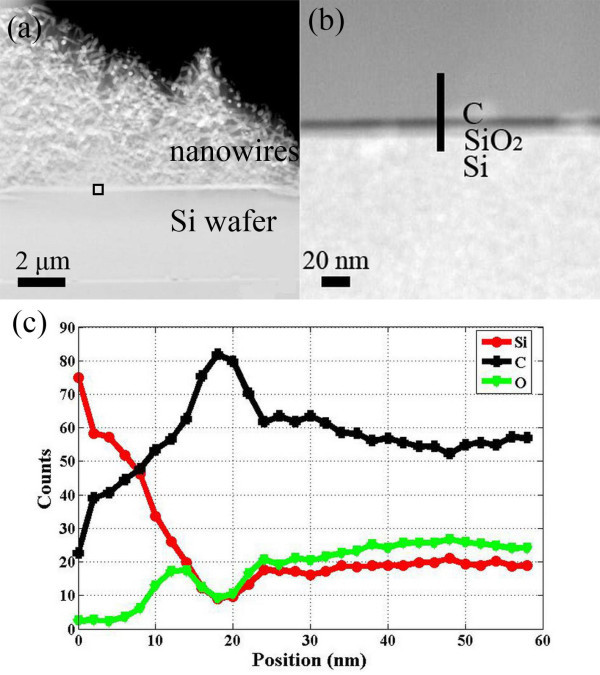
**The HAADF STEM image of the nanowire growing interface**. **(a) **HAADF STEM image of Si-O-N nanowires growing interface. **(b) **An enlarged view of the region marked in (a). **(c) **Elemental counts distribution corresponding to the line in Figure 2b.

Figure 4a shows the HAADF STEM image of the nanowire growing interface. Figure 4b is an enlarged view of the region marked in Figure 4a. Figure 4c shows the elemental counts distribution of the interface corresponding to the line in Figure 4b, where the bottom is defined as the starting point in the horizontal axis, and the line length is 60 nm. It shows that the interface consists of three layers namely Si (0-10 nm), SiO_2 _(10-15 nm), and C (15-25 nm) from the bottom to the top, where SiO_2 _layer is due to the native oxidation of Si substrate, and C layer is formed by sputtering initially.

From the above analysis, a carbon-assisted vapor-solid mechanism is proposed for the growth of Si-O-N nanowires, where carbon has played an important role by introducing a redox reaction with the native silicon oxide layer. The proposed reactions that might have taken place are as follows:(1)(2)(3)

First, this oxide layer partly gets reduced into SiO*_λ _*(1 <*λ *< 2) vapor by the incoming carbon atoms from amorphous carbon film and oxidized carbon (CO), respectively, as explained with the reactions (1) and (2) [19]. Then, the SiO*_λ _*vapor reacts with N_2 _and H_2 _gas into Si-O-N nucleation nanoislands, as shown in reaction (3). The constant reaction on the nanoisland surface would lead to the growth of nanowire arrays.

**Figure 5:**
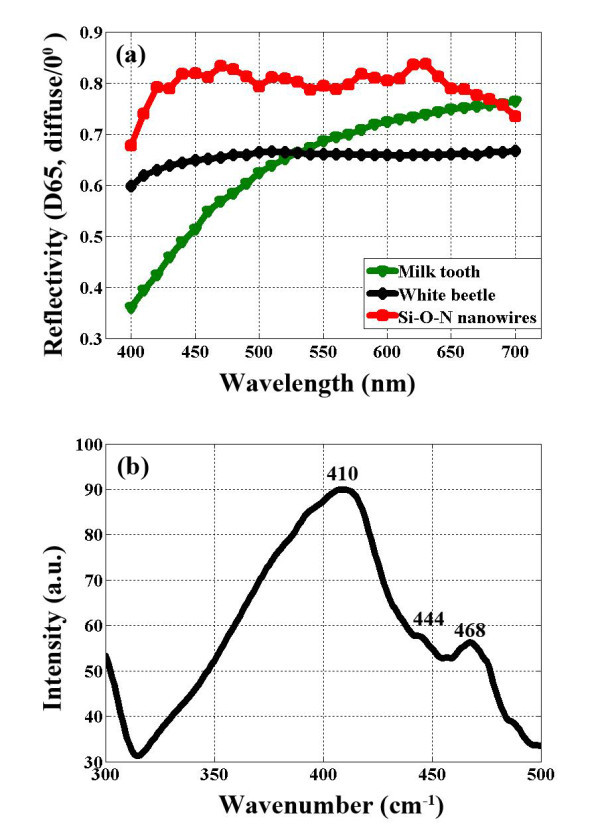
**The optical reflectivity and PL spectra of Si-O-N nanowires**. **(a) **Reflectivity of Si-O-N nanowire mat and its comparison with literature data of human milk tooth and Cyphochilus beetle scale [17]. **(b) **Room temperature PL spectra of Si-O-N nanowires, using a 258-nm line of a Xe lamp as the excitation.

Finally, the optical reflectivity of the obtained Si-O-N nanowire mat in a visible wavelength from 400 to 700 nm was characterized by Datacolor Elrepho photospectrometer, and the result is compared with ultrabright white beetle scale and human milk tooth reported in literature [20]. As shown in Figure 5a, the optical reflectivity of the Si-O-N nanowires is around 80%, while white beetle has an optical reflectivity around 65%. The excellent optical reflectivity of these nanowires is mainly due to scattering of the disordered nanowires. Furthermore, the wide distribution of the 3-nm dark spot in the nanowire leads to the inhomogeneous distribution of refractive index in the nanowire, which may affect the interaction of the incident light with the nanowire and enhance the scattering efficiency. This material may provide a number of potential applications, from cosmetic dopant and white surface for dental implants to energy-saving flat light panels, which need ultrathin reflective backings to scatter the backward light. It will also offer a permeable, flexible, and fault-tolerant layer for diffuse reflector cup, which is in great need for high-power white light-emitting diode (LED) lamps to reduce the optical loss and hence to increase the device efficiency.

The PL spectrum of the Si-O-N nanowires on the Si wafer, taken under excitation with the 258-nm line of a Xe lamp, is presented in Figure 5b. A broad peak ranges from 380 to 500 nm with a maximum centered at 410 nm and two shoulders centered at 444 and 468 nm, respectively. The strong emission around 410 nm arises from recombination either from the conduction band to the N_2_^0 ^level or from the valence band to the N_4_^+ ^level [21]. The weak emission at 444 nm (approximately 2.8 eV) has been experimentally suggested by Noma *et al*. [22], originates from Si-N bonds in Si oxynitride. While the blue PL emission at 470 nm probably has an origin related to Si-O bonds [23].

## Conclusions

In summary, large-scale ultrabrilliant white Si-O-N nanowires were synthesized through carbon-assisted growth. The unique cross-sectional nanostructure of a ternary amorphous nanowire was observed, which might open a new research horizon for growth mechanism of multicomponent nanowires. The nanowires demonstrate extraordinary optical reflectivity in visible wavelength, which will provide new applications in optoelectronic and energy areas such as backlight scattering coating in flat light panels and diffuse reflector for high-power white LED lighting.

## Competing interests

The authors declare that they have no competing interests.

## Authors' contributions

LZ, SX, and DL carried out the fabrication process. XL, WL and TL carried out testing of samples. ZT conceived and designed the experiments. LZ, SX and ZT wrote the manuscript. All authors read and approved the final manuscript.
